# Sequential bilateral testicular tumours presenting with intervals of 20 years and more

**DOI:** 10.1186/1471-2490-13-71

**Published:** 2013-12-09

**Authors:** Klaus-Peter Dieckmann, Petra Anheuser, Florentine Sattler, Tobias Von Kügelgen, Cord Matthies, Christian Ruf

**Affiliations:** 1 Klinik für Urologie, Albertinen-Krankenhaus Hamburg, Suentelstr. 11a, D-22457 Hamburg, Germany; 2 Institut für Hämatopathologie, Fangdieckstrasse 75a, D-22547 Hamburg, Germany; 3 Praxis für Urologie, Rosengarten 5, D-22880 Wedel, Holstein, Germany; 4 Bundeswehrkrankenhaus Hamburg, Abteilung für Urologie, Lesserstrasse 180, D-22049 Hamburg, Germany

**Keywords:** Testicular germ cell neoplasms, Bilateral tumours, Testicular biopsy, Seminoma, Familial germ cell tumours

## Abstract

**Background:**

About 3 – 5% of all patients with testicular germ cell tumour (GCT) develop a contralateral cancer, the majority of which arise within 10–15 years. Little is known about the risk of second GCTs after more than two decades. Here we present 3 cases with very late presenting contralateral GCT and provide a summary of similar cases reported previously.

**Case presentations:**

(1) This white Caucasian man underwent right-sided orchiectomy for a nonseminomatous GCT at the age of 22 years. Additional treatment consisted of retroperitoneal lymph node dissection (RPLND) and chemotherapy with 4 cycles of vinblastin / bleomycin. 36 years later, contralateral seminoma clinical stage 1 developed. Cure was achieved by orchiectomy. Histologically, testicular intraepithelial neoplasia (TIN; intratubular germ cell neoplasia) was detected in the tumour-surrounding tissue.

(2) This white Caucasian male had right-sided orchiectomy for nonseminomatous GCT at the age of 29 years. Pathological stage 1 was confirmed by RPLND. 25 years later, he received left sided orchiectomy for seminoma stage 1. Histologically, TIN was found in the tissue adjacent to seminoma. Two brothers had testicular GCT, too, one with bilateral GCT. (3) This 21 year old white Caucasian man underwent left-sided orchiectomy for nonseminomatous GCT. Pathological stage 1 was confirmed by RPLND. 21 years later, he received organ-preserving excision of a right-sided seminoma, followed by BEP chemotherapy for stage 3 disease. Histologically, TIN was found in the surrounding testicular tissue.

22 cases of bilateral GCT with intervals of 20 or more years have previously been reported, thereof three with intervals of more than 30 years, the longest interval being 40 years.

**Conclusion:**

Apart from increased risks of cardiovascular diseases and non-testicular malignancies, patients with GCT face the specific probability of a second GCT in the long run. This risk persists life-long and is not eliminated by chemotherapy. Contralateral testicular biopsy can identify patients at risk by revealing precursor cells of GCT though false-negative biopsies may occur sporadically. However, in view of the multi-facetted late hazards of GCT patients, this minor surgical procedure might somewhat simplify the long-time care of these patients.

## Background

The phenomenon of malignant growths occurring in both of the testicles has been known for more than a century. Hamilton and Gilbert conducted the first systematic review in 1942. Based on 144 cases collected from the literature they opened their milestone article with the sentence: “Testicular tumors possess a pronounced tendency to bilateralism” [1]. According to recent reviews, 3 – 5% of patients with testicular germ cell tumour (GCT) develop contralateral cancer which corresponds to a 20–30 fold increased relative risk of tumour [2-4]. About 50% of these events occur within 5 years after diagnosis of the primary while 90% do so within a 10 years time-span [5]. Only few more cancers arise thereafter. So far, little is known about the risk of contralateral GCT after very long intervals of more than two decades. All GCTs are preceded by a common precursor named testicular intraepithelial neoplasia (TIN; also called intratubular germ cell neoplasia unspecified, ITGCNU) which is thought to be present in the testicle many years before the clinical manifestation of GCT [6,7]. To identify candidates for second GCTs, contralateral testicular biopsy at the time of orchiectomy of the primary is available [8,9]. However, physicians caring for patients with GCT have widely remained disinclined to this pro-active method of early detection of GCT [10]. Thus clinically, the risk of bilateral testicular tumour needs to be considered during follow-up of patients with GCT. Importantly, as this event may occur beyond the usual 5-years time-span of oncological follow-up, extended observation time is required.

Here we present three cases with exceptionally late onset of second GCT and we provide a survey of the literature regarding the problem of very long intervals between metachronous bilateral testicular tumours.

## Case presentations

### Case #1

This Caucasian white male underwent right-sided orchiectomy for a nonseminomatous testicular GCT at the age of 22 years. History was uneventful; in particular there was neither history of undescended testis nor any prior familial events of testis cancer. Bulky retroperitoneal lymph-node metastases were resected by extended retroperitoneal lymph node dissection (RPLND) followed by four cycles of chemotherapy. As cisplatin was not yet available at that time (1976), the patient received treatment with the contemporary Samuels regimen consisting of vinblastin and bleomycin [11]. Possibly as a sequel of transfusion therapy during multi-modal treatment for GCT, a viral hepatitis B infection was diagnosed one year thereafter. This infection entered a chronic non-aggressive course and the patient stayed disease-free with respect to testicular cancer for 36 years.

**Figure 1:**
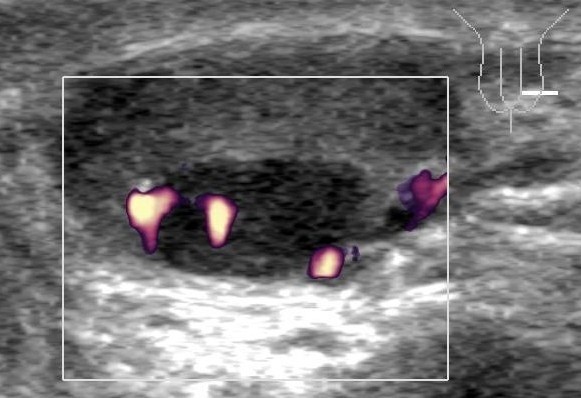
**Grey scale ultrasonography of the testis with colour-coded duplex imaging.** Note the hypo-echoic nodule at the cranial pole of the testis. Positive colour coded duplex signals indicate vascularisation of the tumour. Histologically, this tumour consisted of pure seminoma.

**Figure 2:**
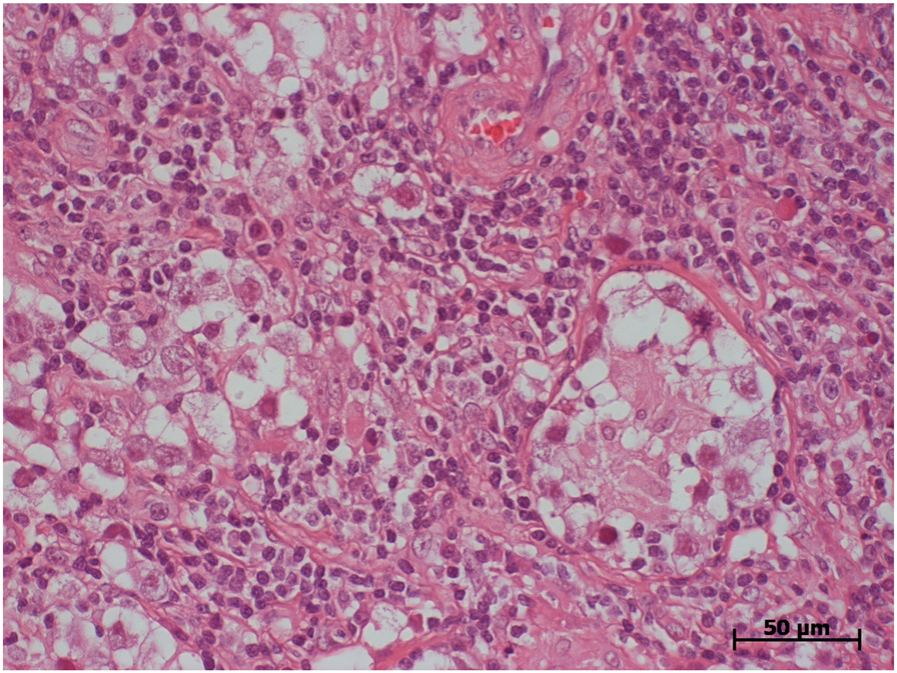
**Histologic section, patient #1. typical histologic features of seminoma.** Note TIN (intratubular germ cell neoplasia) in a seminiferous tubule (right side of picture). Hematoxylin eosin stain; magnification according to scale-bar.

At the age of 58 years, he sought urological advice for an increase of his prostate specific antigen (PSA) level to 1.5 ng/ml. The patient reported no specific symptoms with regard to scrotal disorders. Upon physical examination a firm nodule of about 1 cm in diameter was detected in the left testicle. Serum tumour markers beta Human chorionic gonadotropin (beta HCG), alpha fetoprotein (AFP) and lactic dehydrogenase (LDH) were within normal limits. Scrotal ultrasonography (Figure 1) and magnetic resonance imaging (MRI) revealed a mass of 1.3 cm in diameter within the testicle. As the testicle was small and atrophic, left sided orchiectomy was performed and no attempt of testis-sparing surgery was made. Histological work-up disclosed pure seminoma with vascular invasion but no infiltration of the rete testis. In the seminiferous tubules surrounding the tumour abundant TIN was detected (Figure 2). No metastases were detected, so the patient was placed on a surveillance strategy according to clinical stage I disease. Testosterone substitution is accomplished by periodic intramuscular injections. One year thereafter, the patient is well and without signs of tumour recurrence.

### Case #2

This white Caucasian man underwent right sided orchiectomy for a testicular teratocarcinoma at the age of 29 years. Pathological stage I was ascertained by RPLND. No further therapy was instituted. After 25 years when aged 54 years, the patient discovered a nodule in his remaining testicle by self-palpation. Imaging procedures including ultrasonography, elastography and MRI disclosed a 1.5 cm mass in the testicle. Serum tumour markers beta HCG, AFP and LDH were not increased. As preoperative hormone analysis had revealed a hypogonadal situation with subnormal testosterone level and increased luteinizing hormone (LH), no surgical attempts were made towards a testis-sparing procedure. Instead, a typical inguinal orchiectomy with insertion of a testicular prosthesis was carried out. Histologically, a classical seminoma was identified, with wide areas of TIN in the surrounding tissue. Chest and abdominal CT scans did not reveal metastases. No further treatment was instituted except for hormone replacement therapy. Follow-up of 12 months so far is uneventful. Family history is of particular note: The off-spring consists of one sister and six brothers, two of whom were also struck by testicular cancer. A younger brother succumbed to testicular choriocarcinoma at the age of 23 years. One older brother survived bilateral testicular cancer at ages 23 and 36 years.

### Case #3

This white Caucasian patient underwent left-sided orchiectomy along with a contralateral biopsy for a testicular teratocarcinoma at the age of 21 years. Histologically, the contralateral biopsy was without evidence of TIN. History was uneventful; in particular there was neither history of undescended testis nor any prior familial events of testis cancer. RPLND revealed pathological stage I. Thus, no systemic treatment was applied. At the age of 42 years, he presented with right-sided flank pain. Magnetic resonance imaging revealed dilatation of the right ureter caused by a huge retroperitoneal mass extending from the renal hilum into the pelvis. Beta HCG was slightly increased to 11.9 U/l (reference <1.0 U/l), as was LDH with 400 U/l (reference < 250 U/l), while AFP was within normal limits. Scrotal ultrasonography revealed a mass of 1.9 cm in diameter in the testicle. Organ preserving surgery was performed along with additional biopsies of the adjacent testicular parenchyma. Histological work-up revealed pure seminoma with abundant TIN in the surrounding tissue. Mediastinal lymphadenopathy in addition to retroperitoneal metastases indicated stage III disease with good prognosis according to IGCCCG. The patient was rendered disease-free by systemic therapy with three cycles of cisplatin, etoposide and bleomycin (BEP). Two years thereafter, the patient is relapse-free and well.

## Conclusions

There are at least four lessons to be learnt from these three cases:

(1) The risk of second (contralateral) cancer in GCT patients is pending life-long.

(2) Chemotherapy does not prevent a second testicular tumour.

(3) Familial clustering of testicular cancer appears to be associated with bilateral disease.

(4) Second tumours may arise despite a contralateral biopsy negative for TIN.

According to the most recent review, about 50% of all contralateral testicular tumours arise later than 5 years or more after the primary [2]. In a French analysis, 23% of second tumours occurred between the 10^th^ and the 20^th^ year of follow-up [12], while no case developed later. All of the major investigations on bilateral testicular tumours accord with the contention, that the risk is high during the first ten years and that it is gradually decreasing thereafter forming an asymptotical curve approaching the zero line of risk beyond the 20 years mark [5,13-15]. As a matter of fact, this understanding implicates the sporadic occurrence of such tumours at exceptionally late time points. Accordingly, Table 1 provides a synopsis of 25 cases of the literature (including the present cases) with bilateral testicular germ cell tumours occurring after 20 or more years [1,3,12-14,16-32]. It is of note that already Hamilton and Gilbert in their 1942 review briefly mentioned such a case [1], and Melicow in a scholarly review on testicular new growths did so in 1955 [21]. The longest interval ever reported is 40 years in a Danish man [16]. The present case (#1) is ranking second on this list with an interval of 36 years.

**Table 1:** Sequential bilateral testicular germ-cell tumours with interval of 20 years or more

**Interval**	**Age at first presentation (years)**	**Histology first tumour**	**Second tumour**	**Reference, first author,**	**Year**
40 yrs	40	S	S	Philipsen [16]	1994
36 yrs	22	NS	S	*Present case I*	2013
32 yrs	26	S	NS	Scheiber [17]	1987
31 yrs	31	n.a.	S	Fukuhara [18]	2005
29 yrs	32	NS	S	Manny [19]	1999
28 yrs	n.a.	NS	n.a.	Andreassen [13]	2011
26 yrs	35	NS	NS	Hoekstra [20]	1982
25 yrs	46	n.a.	S	Melicow [21]	1955
25 yrs	25	S	S	Aristizabal [22]	1978
25 yrs	42	S	S	Ohyama [23]	2002
25 yrs	29	NS	S	Klatte [24]	2008
25 yrs	25	NS	S	*Present case II*	2013
24 yrs	n.a.	n.a.	n.a.	Schaapveld [3]	2012
23 yrs	n.a.	S*	S*	Hamilton [1]	1942
23 yrs	23	S	S	Kratzik [14]	1991
22 yrs	n.a.	S	S	Fergusson [25]	1962
22 yrs	32	S	S	Yoshida [26]	1981
22 yrs	22	n.a.	NS	Bach [27]	1983
22 yrs	39	S	S	Celebi [28]	1995
22 yrs	n.a.	n.a.	n.a.	Albers [29]	1999
21 yrs	17	S	S	Tekin [30]	2000
21 yrs	21	NS	S	*Present case III*	2013
20 yrs	n.a.	NS	S	Oesterlind [31]	1987
20 yrs	28	NS	S	Dieckmann [32]	1989
20 yrs	n.a.	n.a.	n.a.	Theodore [12]	2004

S seminoma; NS nonseminoma; n.a. not available.

*most probable histology, assumed from text.

The median age at primary presentation of the patients with very late presenting contralateral testicular tumour is 28.5 years that is not at variance with the over-all median age of patients with germ cell cancer [33]. Yet, there appears to be a slight deviation from the contention that patients with bilateral tumours usually experience a rather early presentation of their primary [12,34,35]. All of our cases had a nonseminomatous histology at first presentation which is somehow at variance with the preponderance of seminoma usually encountered in cases with bilateral testicular GCT [36]. Table 1 does not suggest any association of histology with the particular risk of late second GCT. Little is known about clinical stage at primary diagnosis and during relapse for these particular patients. In most of the reports (Table 1) information on clinical staging is not provided. In our series, only one had metastasized disease at primary presentation while two had stage I disease. So, the basic message of this compilation of cases is the information that the risk of contralateral testicular cancer will persist life-long.

(2) Our first case experienced his second tumour despite interval chemotherapy. Pathogenetically, all GCTs are preceded by the premalignant lesion TIN (ITGCNU) that is believed to be present in the contralateral testicle already at the time of the first tumour. Concerns regarding the low sensitivity of chemotherapy to eradicate the precursor in the contralateral gonad had been raised already in the 1980ies [37]. Accordingly, large series of patients with bilateral testicular tumours clearly revealed that chemotherapy does not protect against second tumours [4,15]. In the largest investigation of bilateral tumours performed on the SEER data base, 39 second tumours (> 1%) were found among 3157 nonseminoma patients receiving chemotherapy [35]. Recently, it was shown that low doses of two cycles of cisplatin-based chemotherapy are probably without any considerable effect on TIN, three or more cycles may eradicate it in about 75% of cases [38]. Our patient had received 4 cycles of chemotherapy, however, that treatment did not involve cisplatin, which clearly is the most efficacious drug. So, one might speculate that the vinblastin/bleomycin regimen could have suppressed TIN only temporarily . Accordingly, it took 36 years for the remaining TIN cells to recover and to finally progress to invasive seminoma. In fact, there is some indication of extended intervals in bilateral GCTs following chemotherapy [13,15]. Our patient apparently represents the case with the longest lag time between sequential bilateral testicular tumours and interval chemotherapy. So, this case represents an exceptional example showing the low efficacy of chemotherapy to prevent sequential testicular neoplasms.

(3) Two brothers of case #2 had suffered from testicular cancer, too, one of whom had even bilateral disease. Approximately 1-3% of all patients with testicular GCT have close relatives with the same diagnosis [34]. Notably, 6-15% of all familial cases of GCT develop bilateral disease while second GCTs occur in only 3-5% among sporadic cases [39]. Accordingly, a strong hereditary predisposition of testicular GCT is assumed [40]. While the clinical evidence for the involvement of genetic factors in the etiology of GCT is undisputed, the assumed Testicular GCT 1 gene has so far not been identified [41]. From a practical point of view, it is concluded that history of familial testicular cancer should increase the clinician´s vigilance regarding the possible development of contralateral cancer. As elucidated by the case, this event may even occur after a very long lag time.

(4) In one of our patients (#3), contralateral GCT developed despite a previous testicular biopsy negative for TIN. False-negative biopsies do occur in less than 10% of cases [8]. Multiple probing has been recommended to reduce the rate of biopsy-failures. In fact, systematic two-site biopsies revealed an 18% extra yield of contralateral TIN [8]. Whether or not double biopsy would have disclosed the true diagnosis at that time remains elusive, but clearly, multiple biopsies are more sensitive than a single one.

Our three cases highlight one of the manifold problems in the long-time follow-up of patients with testicular GCT. The specific risk of a second testicular tumour is one threat in addition to the well-recognized hazards of metabolic disorders, cardiovascular diseases and non-testicular malignancies [42]. The risk of contralateral GCT is not eliminated by chemotherapy. As all of our patients had TIN in the tumour-surrounding tissue, histologically, a contralateral biopsy at the time of the primary might have identified the impending second tumour. Although not explicitly recommended by current guide-lines [43-45], contralateral testicular biopsy is clearly valuable for exploring the particular risk of second testicular cancer [8,46].

## Consent

Written informed consent was obtained from all of the three patients for publication of these Case reports and the two accompanying images. A copy of the written consent is available for review by the Editor of this journal.

## Competing interests

The authors declare that they have no competing interests.

## Authors’ contributions

KPD conceived and designed the study, analyzed the clinical data, and drafted the manuscript. PA performed the ultrasonographic examinations of the patients, participated in the clinical management of cases, participated in designing and of the report and drafting of the manuscript. FS carried out the histological examinations, participated in designing the report. TK participated in the designing of the study, carried out much of the clinical management of the cases, helped analyzing the clinical data. CM participated in the designing of the study, accumulated and abstracted the clinical data, helped in clinical management of the cases. CR conceived the study, participated in designing the report, analyzed the clinical data, and drafted the manuscript. All authors read and approved the final manuscript.

## Pre-publication history

The pre-publication history for this paper can be accessed here:

http://www.biomedcentral.com/1471-2490/13/71/prepub
